# Implementing Primary Prevention into Clinical Practice

**DOI:** 10.1192/j.eurpsy.2025.33

**Published:** 2025-08-26

**Authors:** C. Arango

**Affiliations:** Hospital General Universitario Gregorio Marañon, UCM, CIBERSAM, Madrid, Spain

## Abstract

**Abstract:**

The increasing incidence of mental disorders poses an urgent challenge for psychiatry—one that cannot be addressed by treatment alone. The only sustainable path forward is to **prioritize mental health promotion and primary prevention** at all levels: **universal, selective, and indicated**. While often seen as a long-term investment, primary prevention is **cost-effective** and essential for reducing the future burden of mental illness. This is not science fiction; it is a necessary shift that must happen **now** within our clinical services. In this lecture, I will show how **primary prevention is already being implemented** within the **Institute of Psychiatry and Mental Health in Madrid**, showcasing real-world programs that integrate prevention into everyday psychiatric practice. By embedding prevention into mental healthcare, we can move from a reactive to a proactive model—one that truly addresses the root causes of mental disorders and ensures a healthier future.

**Disclosure of Interest:**

None Declared

